# Unsupervised microstructure segmentation by mimicking metallurgists’ approach to pattern recognition

**DOI:** 10.1038/s41598-020-74935-8

**Published:** 2020-10-20

**Authors:** Hoheok Kim, Junya Inoue, Tadashi Kasuya

**Affiliations:** 1 Institute for Industrial Science, The University of Tokyo, 4-6-1 Komaba, Meguro, Tokyo, 153-0041 Japan; 2 Graduate School of Engineering, The University of Tokyo, 7-3-1 Hongo, Bunkyo, Tokyo, 113-8656 Japan

**Keywords:** Metals and alloys, Characterization and analytical techniques

## Abstract

An efficient deep learning method is presented for distinguishing microstructures of a low carbon steel. There have been numerous endeavors to reproduce the human capability of perceptually classifying different textures using machine learning methods, but this is still very challenging owing to the need for a vast labeled image dataset. In this study, we introduce an unsupervised machine learning technique based on convolutional neural networks and a superpixel algorithm for the segmentation of a low-carbon steel microstructure without the need for labeled images. The effectiveness of the method is demonstrated with optical microscopy images of steel microstructures having different patterns taken at different resolutions. In addition, several evaluation criteria for unsupervised segmentation results are investigated along with the hyperparameter optimization.

## Introduction

**Figure 1:**
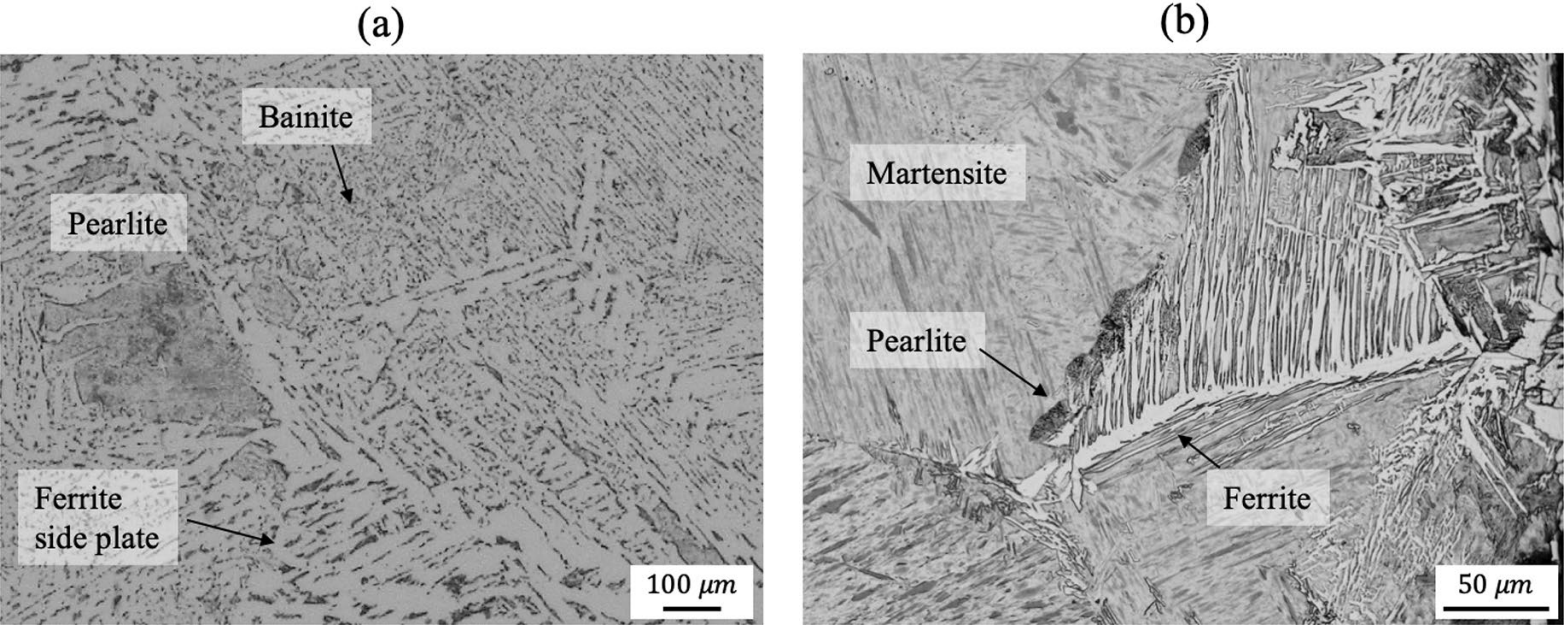
Typical examples of microstructures observed in steel alloys.

A microstructure is a small-scale internal structure of a material, which strongly affects its mechanical, chemical, and electric properties. In particular, steel alloys are known to exhibit a wide range of mechanical properties due to the formation of a wide variety of microstructures such as ferrite, pearlite, bainite, and martensite depending on the cooling process. Figure 1 depicts typical examples of microstructures observed in steel alloys. A difference in microstructure results in different mechanical properties^1^. Dual-phase steel, which consists of a ferrite matrix containing hard martensitic islands and is widely used in the automobile industry, is a good example of this phenomenon. A higher yield strength can be achieved not only by increasing the volume fraction of the hard martensitic microstructure^2^ but also by modifying the microstructural morphology^3^. Accordingly, characterizing the microstructures of steel is an important task for the development of advanced high-strength steels. However, this is very challenging because the microstructure usually consists of one or more phases that are not easily distinguishable. Conventionally, the phases of steel alloys have been classified by the manual analysis of light optical microscopy (LOM) or scanning electron microscopy (SEM) images^4^. However, this approach has the drawback that it requires a labor-intensive pixelwise classification performed by experienced experts. Therefore, approaches based on machine learning algorithms have attracted great attention due to their efficiency. For example, Choi et al.^5^ introduced a classification algorithm based on support vector machines (SVM)^6^ for detecting defects on the surface of a steel product. Further applications of SVM were presented by Gola et al.^7,8^, where the microstructures of steel alloys were classified into constituent phases. Another frequently applied technique is the random forest, which is a classification algorithm composed of multiple decision trees^9^. Various studies have found that steel microstructures can be accurately classified using random-forest-based methods^10–12^.

Nowadays, one of the most emerging microstructure classification schemes is a deep learning based approach. Deep learning is also a class of machine learning algorithms that use multiple processing layers to learn representations of the raw input^13^. These multilayers are called neural networks and are being actively applied to microstructure classification tasks. de Albuquerque et al.^14,15^ first proposed the application of artificial neural networks to classify and quantify simple nodular microstructures from cast iron images. More recently, convolutional neural networks (CNN) have been intensively used in the field of computer vision owing to their fast and efficient classification performance^16^. Azimi et al. reported the successful implementation of VGGNet^17^, which is a pretrained CNN proposed by Krizhevsky, for classifying microstructures from LOM and SEM images of a steel^18^. Since then, a number of studies applying CNN have been conducted such as the application of DenseNet^19^ to detect defects in steels^20^ and ResNet18^21^ to classify microstructures of welded steels^22^. It was verified that the performance of CNN-based methods is as good as that of humans.

Deep learning algorithms are trained with a vast number of labeled images so that they can learn how features are related to the target and this scenario is referred to as supervised learning. However, there are several difficulties when applying supervised learning algorithms to microstructure classification. First, microstructures are not easily distinguishable even for experienced experts, so the preparation of a large labeled dataset is extremely labor-intensive and time-consuming. Second, the size of the input image is limited when using pretrained parameters. For example, the width and height of the input image should be fixed to 224 $\times$ 224 pixels when implementing well-known networks such as VGGnet^17^, DenseNet^19^, and ResNet18^21^.

In contrast, human researchers perceptually distinguish different microstructures from various patterns under various illumination conditions without the need for labeled images. In view of the fact that metallurgists can identify different microstructures hidden in a single micrograph even at first glance, we design an unsupervised segmentation method for low-carbon steels by mimicking the way in which metallurgists investigate each micrograph. The algorithm is strongly motivated by the one proposed by Kanezaki^23^ and is based on CNN accompanied by a superpixel algorithm. Segmentation results demonstrate that the proposed method can be used to distinguish microstructures. In addition, the quality of the segmented images is assessed using evaluation criteria for unsupervised segmentation scenarios.

## Methods

**Figure 2:**
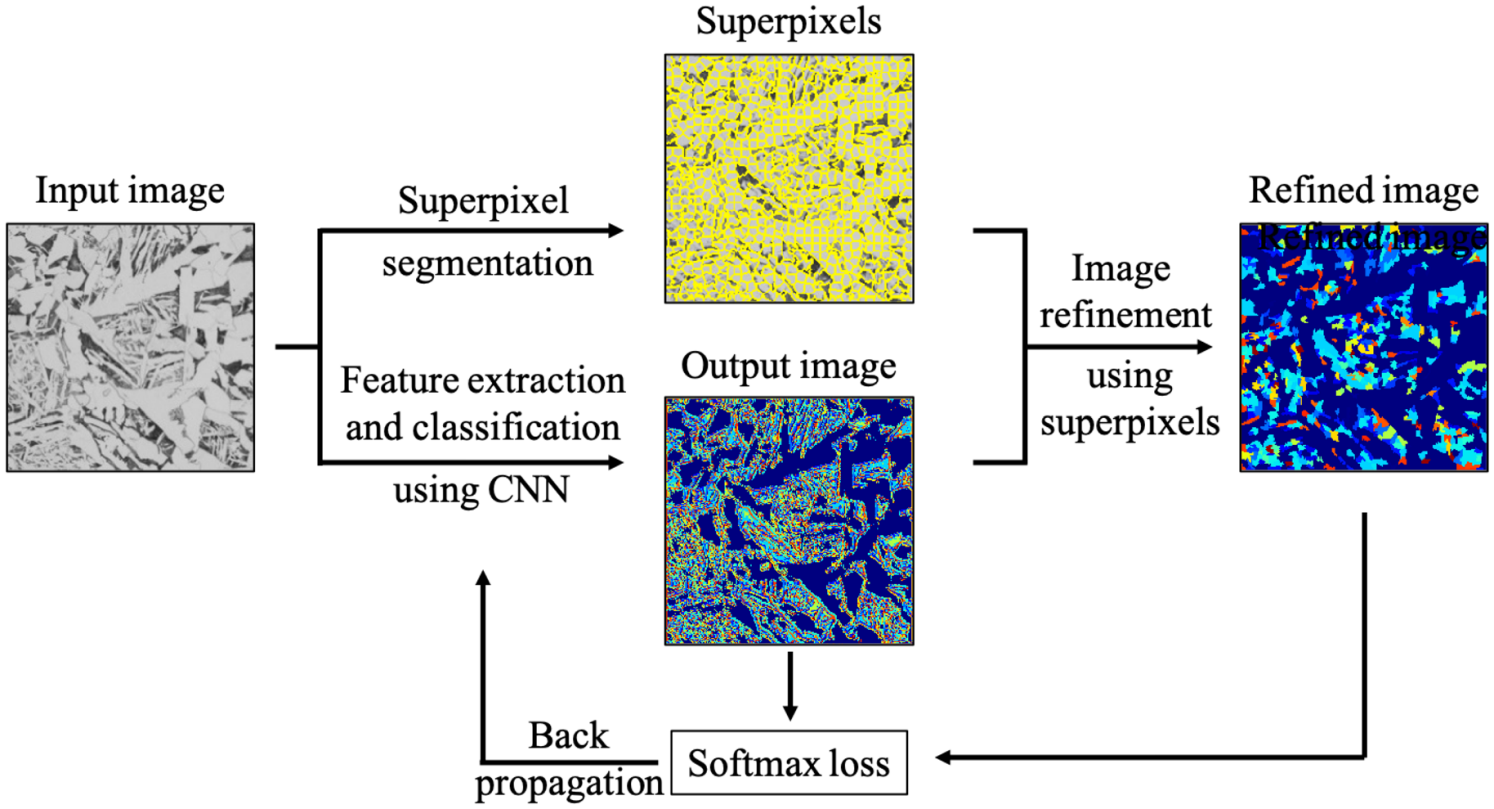
Schematic illustration of the applied algorithm composed of CNN and a superpixel algorithm.

The approach to identifying hidden microstructures in a single image adopted by metallurgists generally consists of three steps; at first, the whole image is roughly subdivided into many small regions of interest (ROIs) with identical contrast and texture characteristic length, then microstructural features representing each ROI are searched. Finally, ROIs are grouped into several classes that have some similarity with respect to the derived microstructural features. Accordingly, the unsupervised segmentation algorithm is designed as follows. After a single micrograph is input, it first undergoes superpixel segmentation to acquire small ROIs with identical contrast and texture characteristic length. Then, a CNN computation is carried out to derive a feature representing each ROI. The network parameters are trained so that the feature with the highest frequency in each ROI dominates the other features in the region. In addition, by applying the same CNN computation to all the superpixels, several features commonly appearing in several different ROIs are selected automatically. In other words, connected pixels with similar colors and other low-level properties, such as hue, luminance, and contrast, are grouped and assigned the same label by the superpixel computation and the spatially separated groups having similar textures are assigned the same label by CNN computation. By combining them, a group of pixels having similar features can be categorized into the same cluster and a schematic of the network is given in Fig. 2.

The following subsections provide detailed explanations of the algorithms employed in each step of the unsupervised segmentation method. First, the fundamental knowledge of the CNNs is given in order of the computation procedure. Then, an explanation of the superpixel algorithm is presented with sample images. Finally, several evaluation criteria are addressed to assess the results of segmentation without labeled images.

### CNN

**Figure 3:**
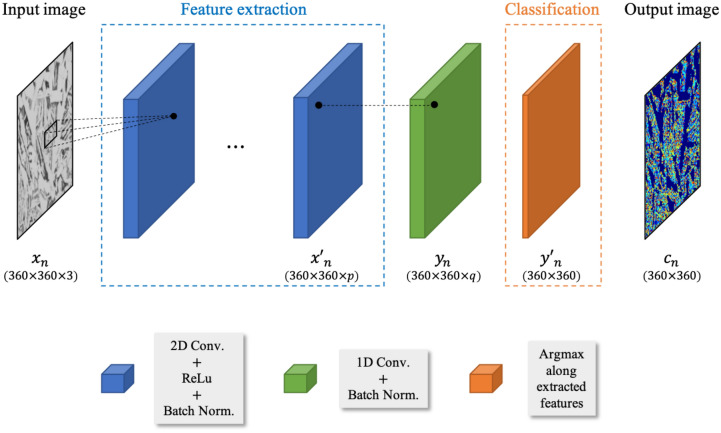
Architecture of the algorithm with an image size of $360\times 360\times 3$ and detailed information about the layers of the CNN network.

The structure of the CNN used in this study consists of an input layer, hidden layers, and an output layer as illustrated in Fig. 3. The input layer accepts the external image data and the output layer gives the predicted answer computed by the network. The computation is mainly conducted in hidden layers consisting of one or more convolutional filters (or kernels) that are indispensable building blocks of the CNN. Filters are typically composed of a set of learnable parameters and perform 2D filtering on input images by conducting the linear operation

1 $${h}_{n}=\left({W}_{m}\otimes{x}_{n}\right)+{b}_{m}$$

${\left\{{x}_{n}\right\}}_{n=1}^{N}$ is a set of $p$-dimensional feature vectors of image pixels, where $N$ denotes the total number of pixels in images. ${W}_{m}$, ${b}_{m}$, and ${h}_{n}$ are respectively trainable weights of the filters, bias, and feature map obtained after convolutional operation $\otimes.$ An activation function is followed by each convolutional layer in order to introduce nonlinearity into the neural network. One of the widely used functions is the rectified linear unit (ReLU) mathematically expressed as

2 $$f\left({x}_{n}\right)=\left\{\begin{array}{l}{x}_{n} \quad for\,\, {x}_{n}>0,\\ 0\quad for\,\, {x}_{n}\le 0.\end{array}\right.$$

Then, additional layers for batch normalization, which is a technique recently proposed by Ioffe and Szegedy^24^, are connected to the activation function. The idea is to normalize the outputs of the activation function so that a subsequent convolutional layer can receive an image having zero mean and unit variance as

3 $${x}_{n}^{^{\prime}}=\frac{{x}_{n}-{\stackrel{-}{x}}_{n}}{\sqrt{{\sigma }^{2}\left({x}_{n}\right)+\epsilon }},$$

where $\stackrel{-}{x}$, ${\sigma }^{2}\left(x\right)$, and $\epsilon$ are respectively the mean of $x$, the standard deviation of $x$, and a constant to provide numerical stability whose value is usually set as $1\times {10}^{-5}$. It has been reported that rescaling the image before inputting allows a faster, efficient, and more stable learning^25^. The following layer is a linear classifier layer ${\left\{{y}_{n}\right\}}_{n=1}^{N}$ that categorizes the obtained features of each pixel into $q$ classes. The linear relationship is applied in this study:

4 $${y}_{n}={W}_{c}{x}_{n}^{^{\prime}}+{b}_{c},$$

where ${W}_{c}$ and ${b}_{c}$ are the weights of the 1D convolution filters and bias, respectively. After normalizing ${y}_{n}$ so as to obtain ${y}_{n}^{^{\prime}}$, the argmax classification is applied to choose the features with the maximum ${y}_{n}^{^{\prime}}$. Finally, a 2D output image having segmentation classes ${c}_{n}$ is obtained.

### Superpixel segmentation

**Figure 4:**
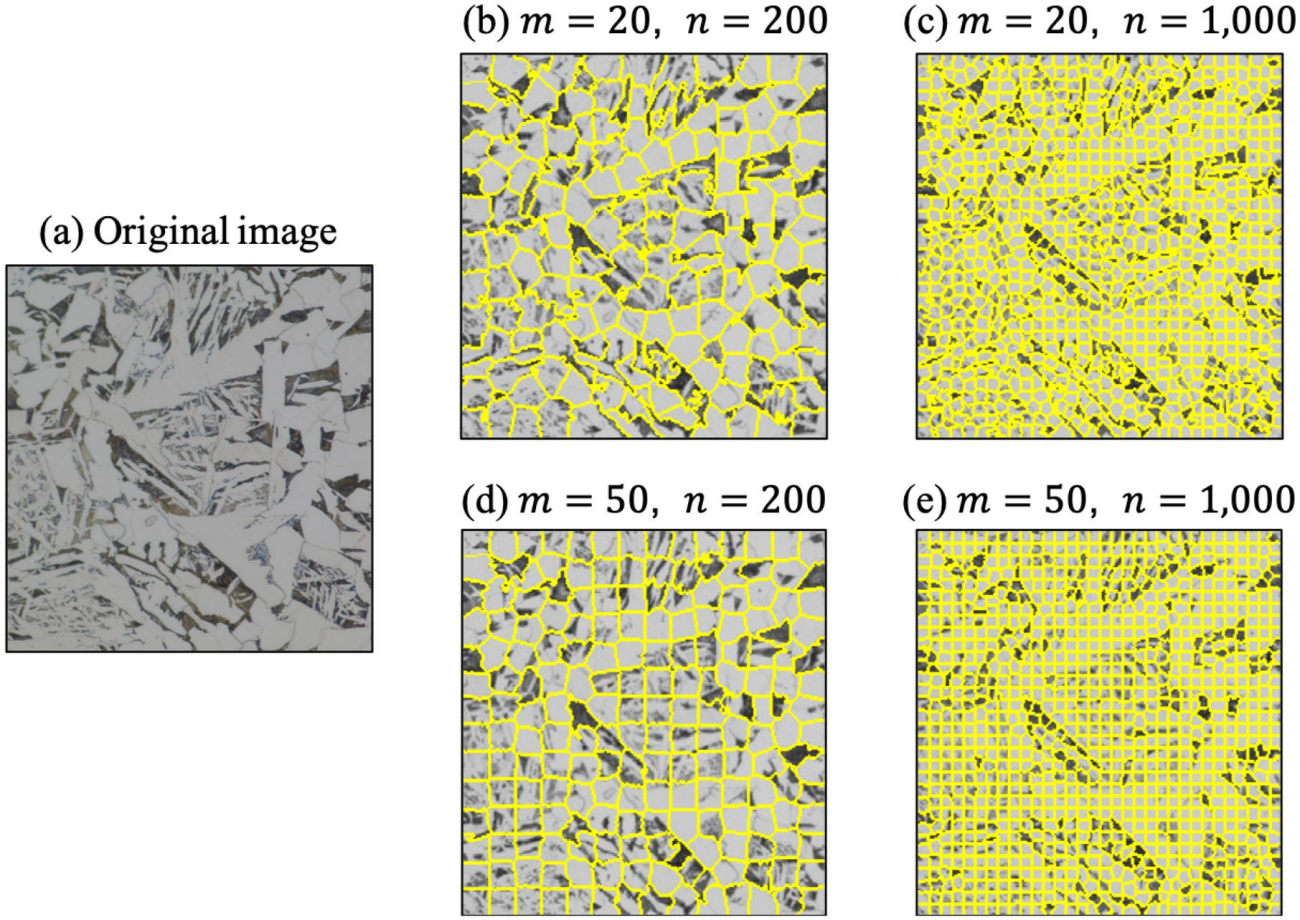
Effects of the compactness m and the number of superpixels n on superpixel segmentation: **(a)** original image, **(b–e)** superpixel segmentation results with various m and n.

When a human distinguishes microstructures of steels, similar microstructures are generally grouped based on the basis of colors. For example, if two regions have the same color, then they will be allocated the same class. In addition, spatial characteristics or morphologies are considered when dividing regions. Similarly, a superpixel algorithm distinguishes different regions called superpixels, which are regions of continuous pixels having similar characteristics such as pixel intensity, by considering the color similarity and spatial proximity. Thus, it provides a convenient and compact representation of images when a human performs classification tasks. The simple linear iterative clustering (SLIC) algorithm^26^ is introduced in this study among the various algorithms for clustering superpixels^27–29^. There are two important hyperparameters when obtaining superpixels using this SLIC algorithm. The first is the number of superpixels, which defines the number of regions in the input image. The second is the compactness factor $m$, which balances the color proximity and spatial proximity. The lower the value of $m$, the more color proximity is emphasized. Its effect on the clustering is illustrated for a steel microstructure image in Fig. 4. Readers are recommended to refer to the literature^26,30^ for further information about the concept and implementation of the SLIC algorithm.

### Training of the CNN

Regarding the training of the CNN, the softmax function is used as a loss function, which measures the difference between the CNN output image and the refined image. The parameter tuning of the CNN is resolved using the stochastic gradient descent (SGD) optimization algorithm and backpropagation. Regarding SGD hyperparameters, it is empirically known that a learning rate of 0.1 and a momentum of 0.9 generally gives good results. Finally, the training is set to be finished after the maximum number of iterations is reached or the number of segmented regions becomes less than the number of classes set by a user.

### Evaluation criteria

When evaluating a segmentation result, two approaches are generally taken. First is a supervised evaluation method which compares a segmented image with a labeled image. Even though a quantitative comparison can be easily done, it requires a manually labeled reference which is intrinsically subjective and labor intensive.

Another common alternative is an unsupervised evaluation method where the quality of a result is assessed based solely on segmented results. It enables the objective evaluation without requiring a manually labeled reference image. In this study, the performance of unsupervised segmentation with an $N\times M$ image size is evaluated using the $F$ ^31,32^$F{^{\prime}}$, and $Q$ ^32^ metrics respectively given by Eqs. (5–7):

5 $$F= \frac{1}{1000\left(N\times M\right)}\sqrt{R}\sum_{i=1}^{R}\frac{{e}_{i}^{2}}{\sqrt{{A}_{i}}}$$

6 $$F{^{\prime}}= \frac{1}{1000\left(N\times M\right)}\sqrt{\sum_{A=1}^{Max}{\left[R\left(A\right)\right]}^{1+1/A}}\times \sum_{i=1}^{R}\frac{{e}_{i}^{2}}{\sqrt{{A}_{i}}}$$

7 $$Q= \frac{1}{1000\left(N\times M\right)}\sqrt{R}\sum_{i=1}^{R}\left[\frac{{e}_{i}^{2}}{1+log{A}_{i}}+{\left(\frac{R\left({A}_{i}\right)}{{A}_{i}}\right)}^{2}\right].$$

$R$, ${A}_{i}$, and ${e}_{i}^{2}$ are the number of segmented regions, the area of the $i$th region, and the average color error of the $i$th region, respectively. With these criteria, the segmentation result with the lowest value is preferred. The basic concept of the metrics is to assess the quality of segmented images by comparing average color error values ${e}_{i}$ of segmented regions of input and output images. In addition to the goodness of fit of the color, terms including $R$ are introduced in order to penalize segmentations that form too many regions. Zhang et al.’s article^33^ is recommended for readers interested in a more detailed explanation about the evaluation of unsupervised image segmentation.

### Implementation details

For the computational environment, a Tesla V100 NVIDIA GPU was used with the PyTorch framework and CUDA platform. The number of convolutional layer, filters in each convolution layer $p$ and the linear classifiers $q$ were set as 2, 100 and 50, respectively. For the SLIC superpixel algorithm, the compactness and the number of superpixels were defined as 20 and 50,000, respectively. The time taken for the unsupervised segmentation and the $F$ value evaluation with a 1000 $\times$ 1000 pixel image was about 30 s and less than 1 s, respectively.

## Results and discussions

### Dataset

To obtain the input image data, we prepared low-carbon steel samples whose composition is presented in Table 1, which were polished and etched using 5% picral + 0.5% nital solution. Then, optical micrographs were taken at different magnifications. It was confirmed that each image consists of various microstructures such as grain boundary ferrite, ferrite side plate, pearlite, bainite, and martensite. The obtained image dataset was used as input data for training the network without modifying the image size.

**Table 1:** Chemical composition (wt. %) of the low-carbon steel used in this work.

C	Si	Mn	P	S	Al	N	O
0.152	0.015	1.51	0.007	0.0016	0.026	18 (ppm)	28 (ppm)

### Segmentation

**Figure 5:**
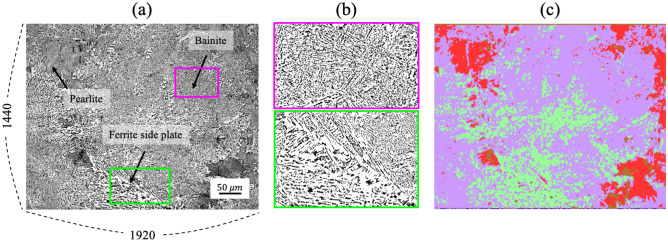
**(a)** Input microstructure image, **(b)** zoomed images showing bainite (upper row) and ferrite side plate (lower row) phases, and **(c)** segmentation result with colors indicating different classes.

Figure 5a shows one of the low-magnification micrographs with 1920 $\times$ 1440 pixels used for the input data. There are different microstructures: ferrite side plate, pearlite, and bainite. Figure 5b shows the zoomed images of the ferrite and bainite. It is clear that their morphological characteristics differ from each other in that bainite generally has finer microstructure than ferrite and includes finer carbide precipitates. However, both microstructures appear in white with small isolated dots distributed inside them. As they have the same color, traditional approaches, which rely merely on the image contrast, have encountered difficulty in distinguishing these microstructures. The segmentation result obtained using the present method is given in Fig. 5c, in which ferrite side plate, pearlite, and bainite are colored green, red, and purple, respectively. It is demonstrated that ferrite, bainite, and pearlite are well segmented. This result suggests that microstructures with similar contrasts but different morphologies can be divided on the basis of their morphological features using the present method. It also implies that the method can be effectively applied to distinguish the microstructures in an image taken at a lower magnification.

**Figure 6:**
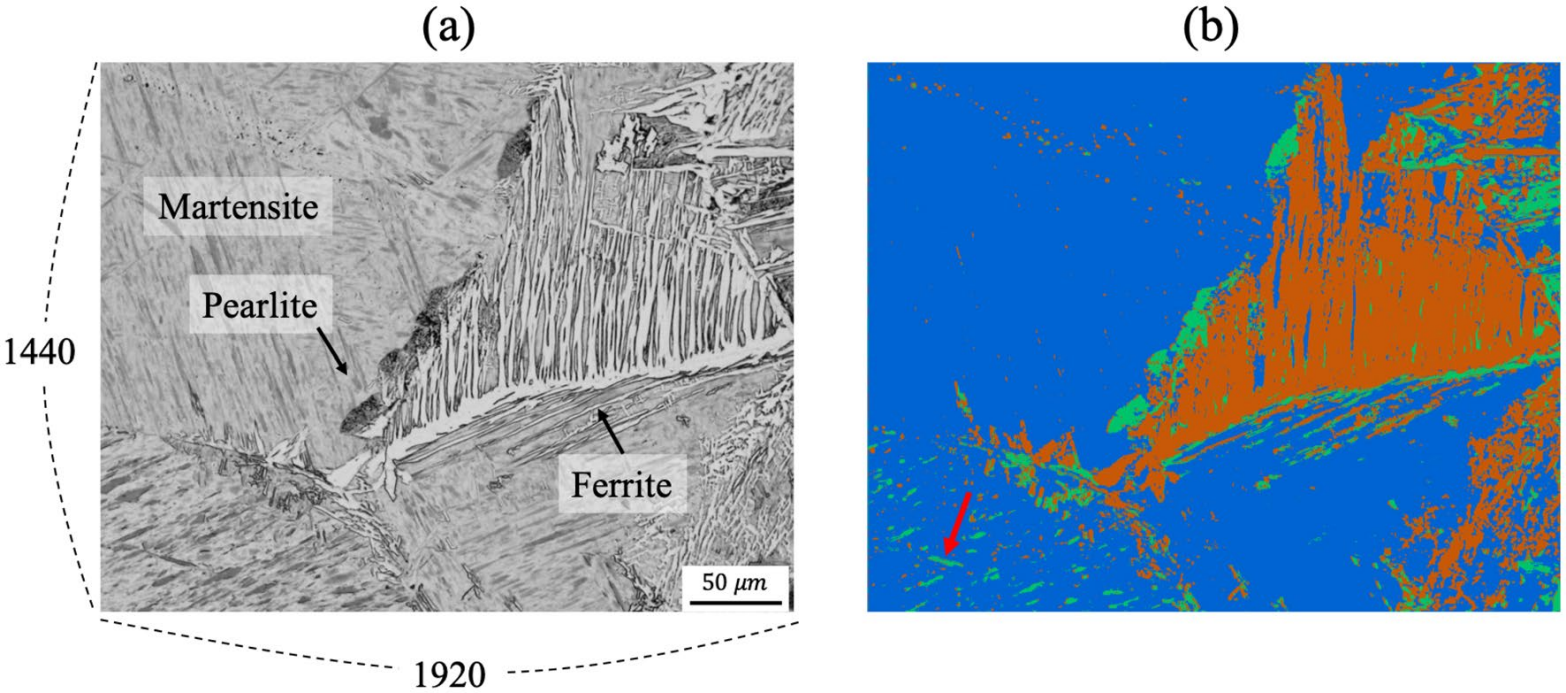
**(a)** Highly magnified input microstructure image and **(b)** segmented image represented by various colors indicating different classes.

Next, unsupervised segmentation is tested for microstructures observed at a higher magnification of 1920 $\times$ 1440 pixels. The input image shown in Fig. 6a consists of four different microstructures: grain boundary ferrite and needle-shaped ferrite side plate in white, pearlite in black, and martensite as the background. The result of unsupervised segmentation is shown in Fig. 6b, where ferrite, pearlite, and martensite are colored in orange, green, and blue, respectively. The result indicates that ferrite, pearlite, and martensite are well distinguished pixelwisely. However, some spots in the lower left part of Fig. 6b (marked with a red dotted circle) were recognized as a pearlite region because they are also displayed in black. Nevertheless, the overall segmentation was successful considering the complexity of the input microstructure.

**Figure 7:**
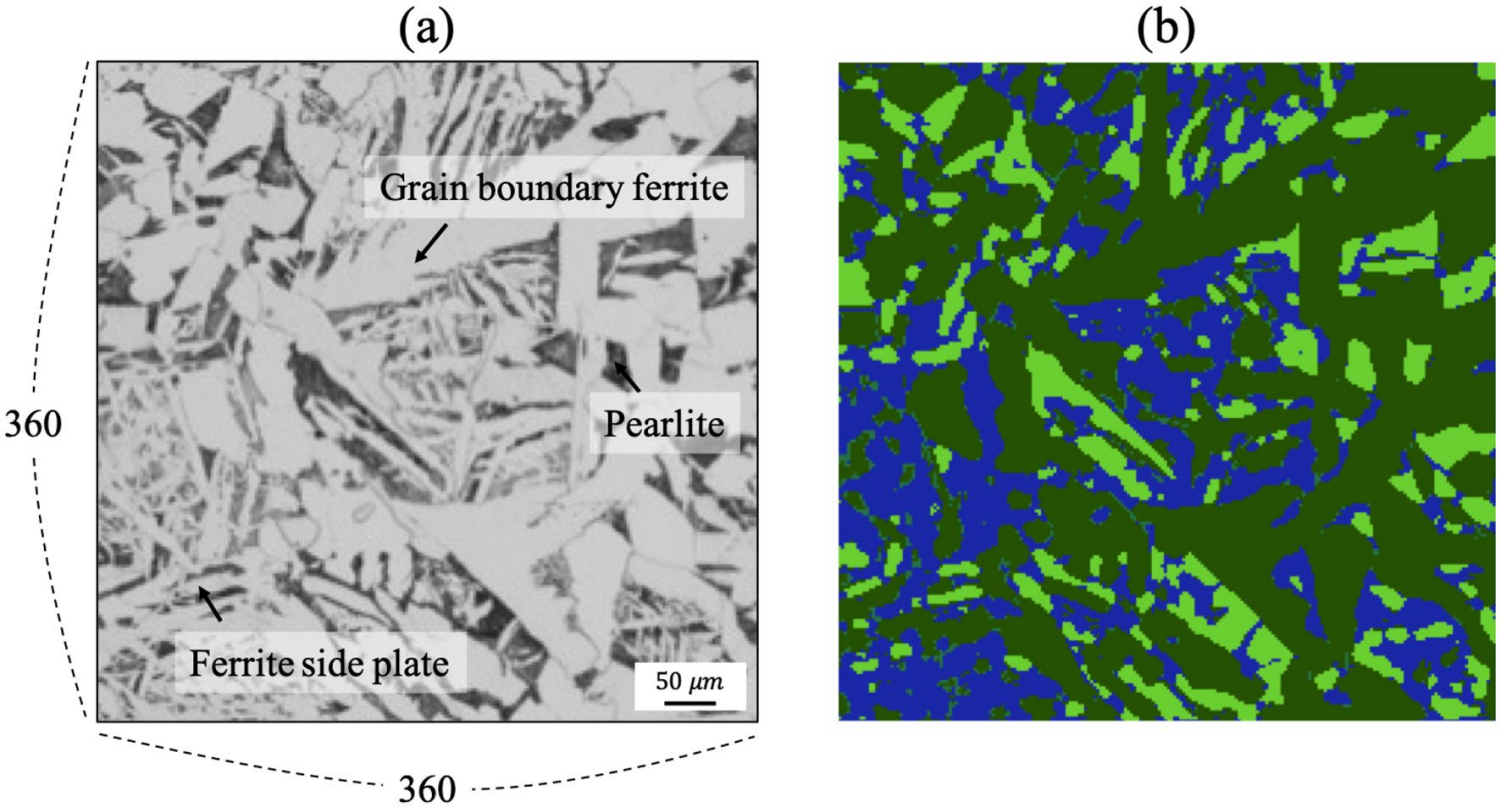
**(a)** Low resolution input microstructure image and **(b)** segmented image represented by various colors indicating different classes.

Lastly, the segmentation was conducted for a micrograph with a much lower resolution. Figure 7a shows the input micrograph of a typical low-carbon microstructure with only 360 $\times$ 360 pixels. The microstructure is composed of grain boundary ferrite, ferrite side plate, and pearlite. Figure 7b shows the segmentation result with grain boundary ferrite in green, ferrite side plate in blue, and pearlite in light green. Unlike the previous cases, the segmented areas have rounded corners. This might be caused by the lack of information due to the low resolution. Other than that, the overall segmentation appeared to be successful even with the limited resolution.

**Figure 8:**
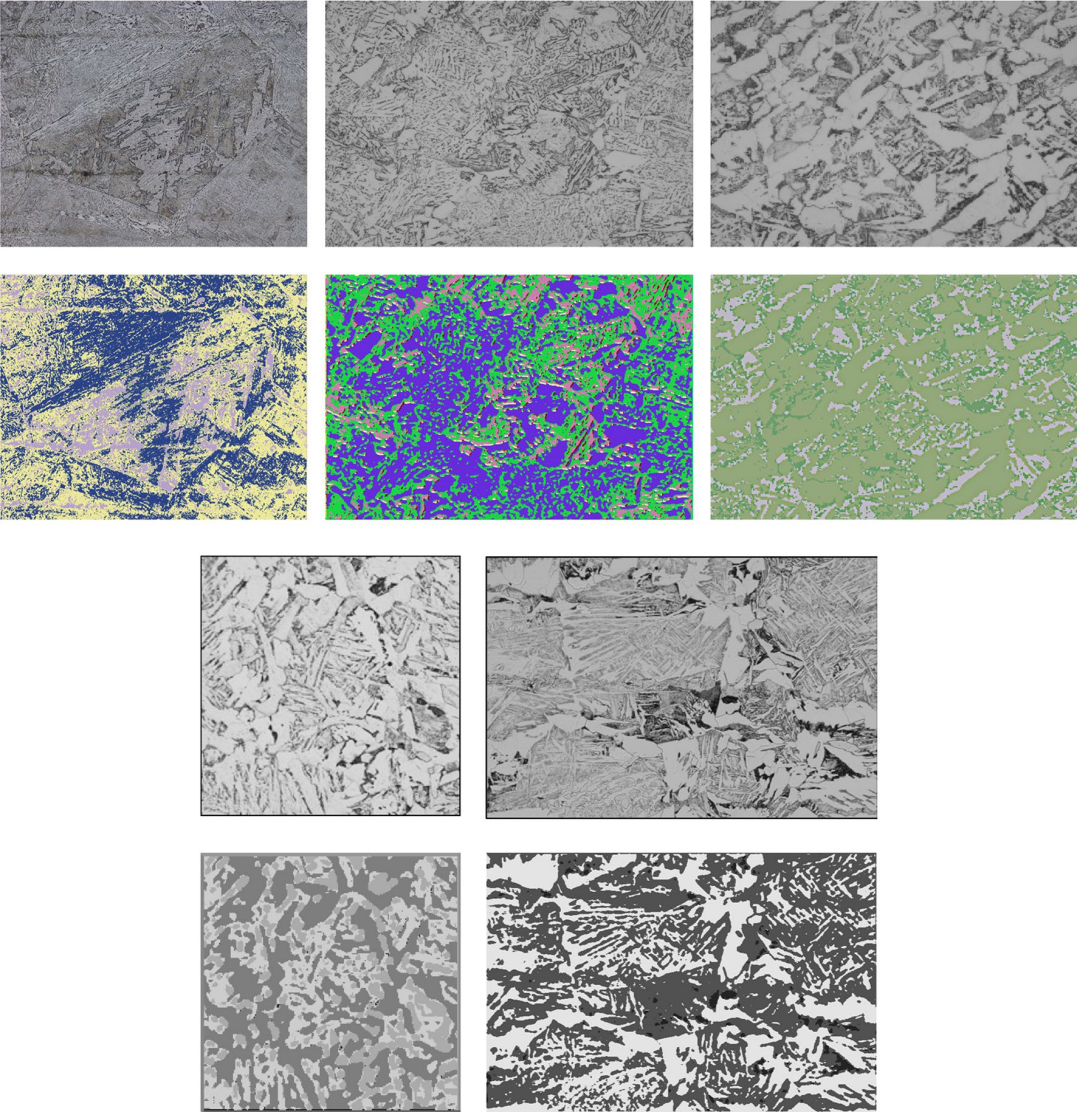
Examples of microstructure images (upper row) segmentation results (lower row).

In addition to the aforementioned segmentation results, other results for low-carbon steels with various microstructures are given in Fig. 8. They also indicate that the performance of the present method was successfully demonstrated.

### Evaluation

**Figure 9:**
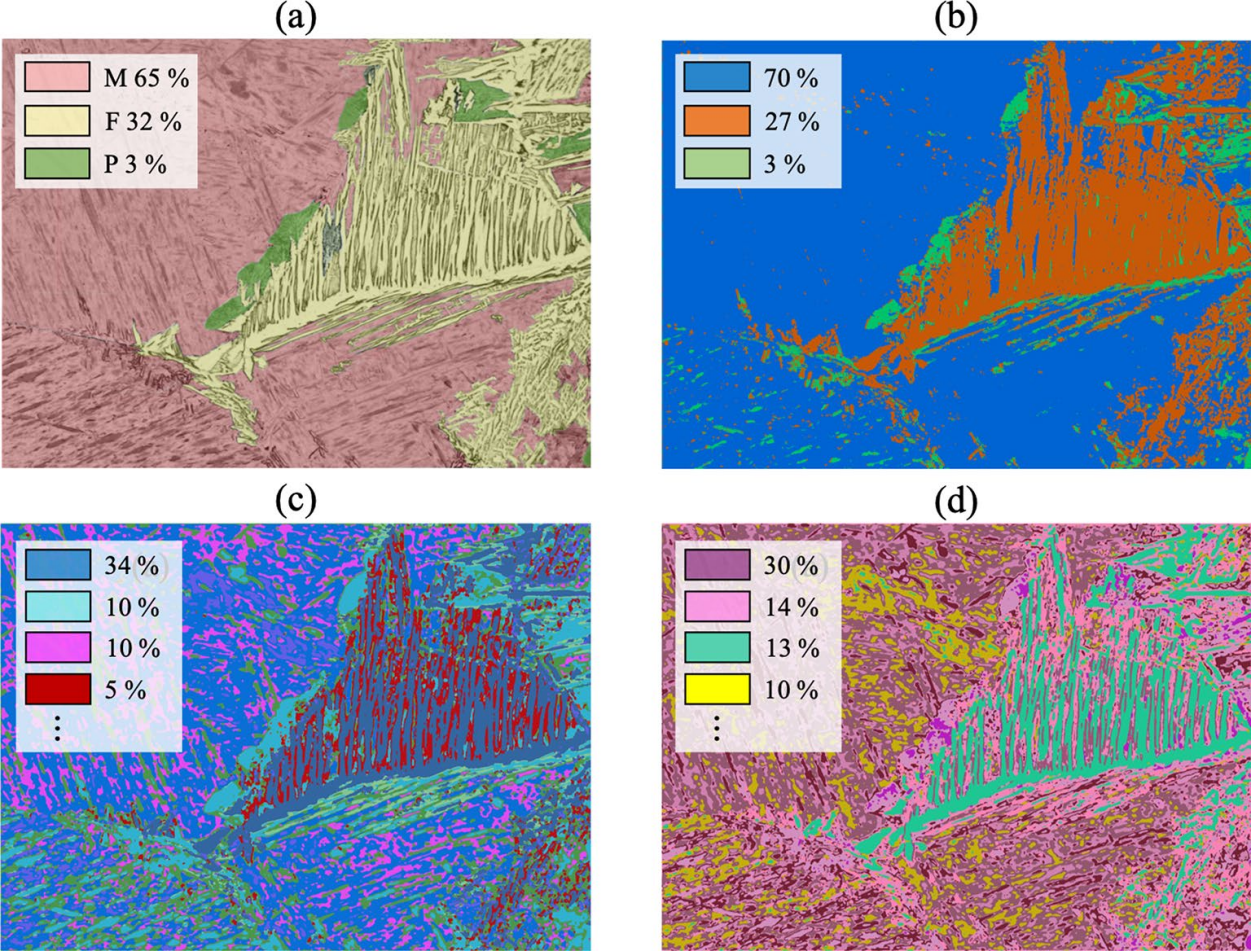
Comparison of **(a)** the labeled image consists of martensite (M), ferrite (F), and pearlite (P) phases created by manual classification and the segmented images with different hyperparameter settings for the number of classes; **(b)** 3 classes, **(c)** 10 classes, and **(d)** 20 classes.

Figure 9 shows an example of the comparison between manual and unsupervised segmentation results along with the fraction of their constituent areas. The image labeled by our experienced experts indicates that the microstructure is composed of three different phases; 65% of martensite (M), 32% of ferrite (F), and 3% of pearlite (P). Likewise, the fraction of constituent regions of the segmented images obtained for three different numbers of classes are given in Fig. 9b–d. In the unsupervised segmentation results, the each region is not classified into a certain category. However, It is easily noticeable that the result consists of three classes (Fig. 9b) is consistent with the reference image in terms of the segmentation boundary. In addition, the fractions of the phases are in good agreement with the manual analysis. It is confirmed that the accuracy of the proposed unsupervised segmentation method is comparable to that of the previously reported supervised method^10^.

**Figure 10:**
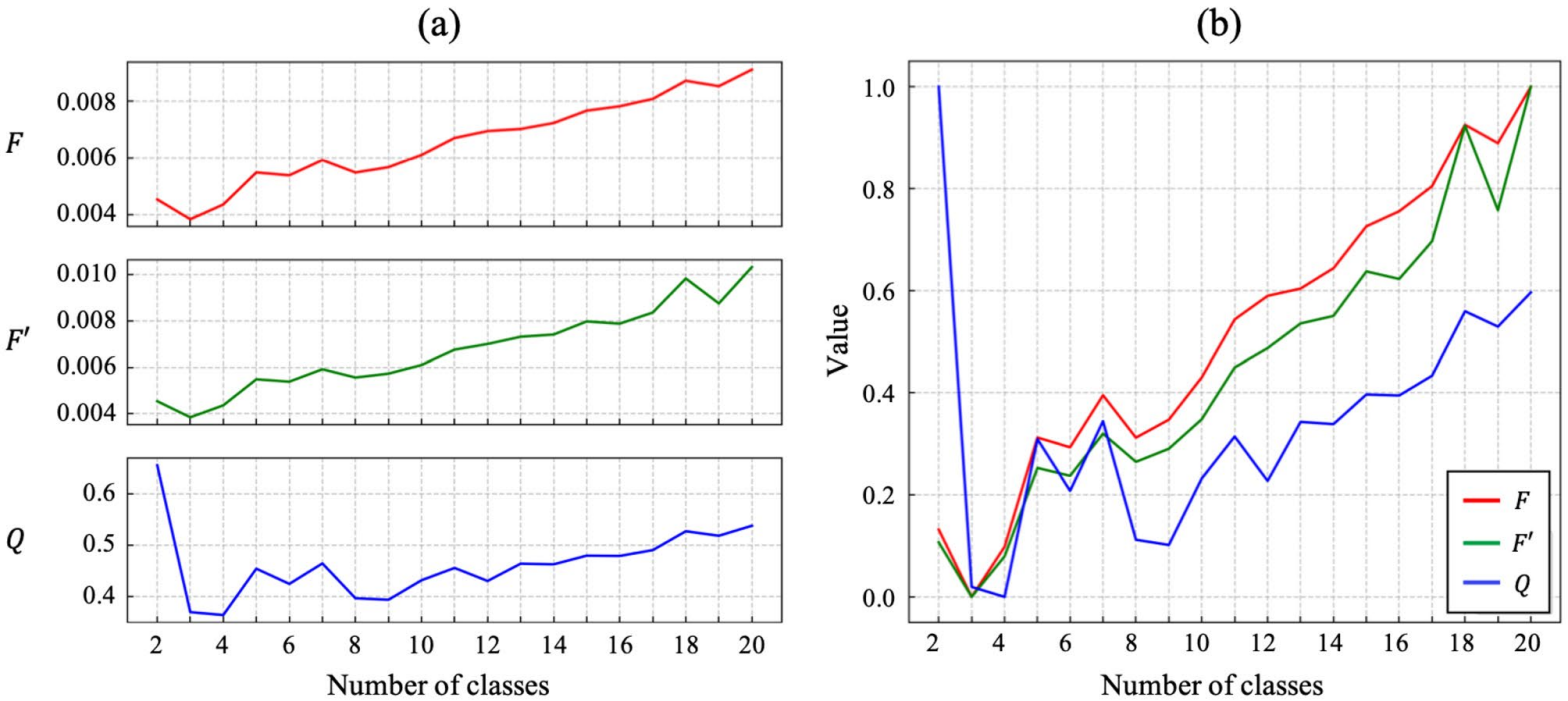
**(a)** Estimated F, F, and Q values for segmentation results with different numbers of classes and **(b)** their corresponding values normalized to the range [0, 1].

Since there can be numerous possible segmentation results for one microstructure if a hyperparameter, such as number of classes, is varied as clearly demonstrated in Fig. 9. Therefore, we need a measure to clarify which of the parameters is the most appropriate among given results. Since this deep learning method produces a different result each time owing to the randomness occurring for various reasons such as the weight initialization, each of the unsupervised evaluation criterion is averaged over ten times repetition for the evaluation of the different segmentation results. Figure 10a gives the evaluation results for Fig. 6a with increasing number of classes using the three unsupervised segmentation evaluation criteria. $F$ and $F^{\prime}$ are lowest when there are three classes. This means that the microstructure is better to be divided into three regions. As the number of classes increases, the values also increase, meaning that quality of the result deteriorates. On the other hand, $Q$ shows its minimum when there are four classes. In addition, it was observed that the $Q$ with two classes is much higher than $F$ and $F^{\prime}$. This is because $Q$ was designed to give a high penalty for regions with a large area having a very little variation in color^33^. Therefore, when there were only two regions which inevitably include various colors, $Q$ was higher.

Even though these criteria provide an easy way to evaluate the segmentation result, it is still difficult to compare criteria since their scales are different. In order to compare these criteria, their values were normalized to the range [0, 1] as shown in Fig. 10b. In addition, they were ranked according to their values as given in Table 2 in order to compare them with each other more easily. Note that normalization does not affect the rankings estimated by these evaluation criteria. A noticeable difference is that segmentation result with nine classes marked the third rank with $Q$ criterion, which is not preferred with other criteria. As mentioned previously, $Q$ disfavors large areas with various colors and prefers small areas with homogenous colors. Therefore, a larger number of classes is likely to be chosen with $Q$ than with $F$ and $F^{\prime}$. Through these results, it was concluded that $F$ is the most appropriate criterion for evaluating the unsupervised segmentation of a steel microstructure.

**Table 2:** Numbers of classes and their values arranged in order of the rank determined from the evaluation criteria.

Rank	1st	2nd	3rd	4th	5th
$F$	3 (0)	4 (0.10)	2 (0.13)	6 (0.30)	5 (0.31)
$F{^{\prime}}$	3 (0)	4 (0.08)	2 (0.11)	6 (0.24)	5 (0.25)
$Q$	4 (0)	3 (0.02)	9 (0.10)	8 (0.11)	6 (0.21)

### Hyperparameter optimization

**Figure 11:**
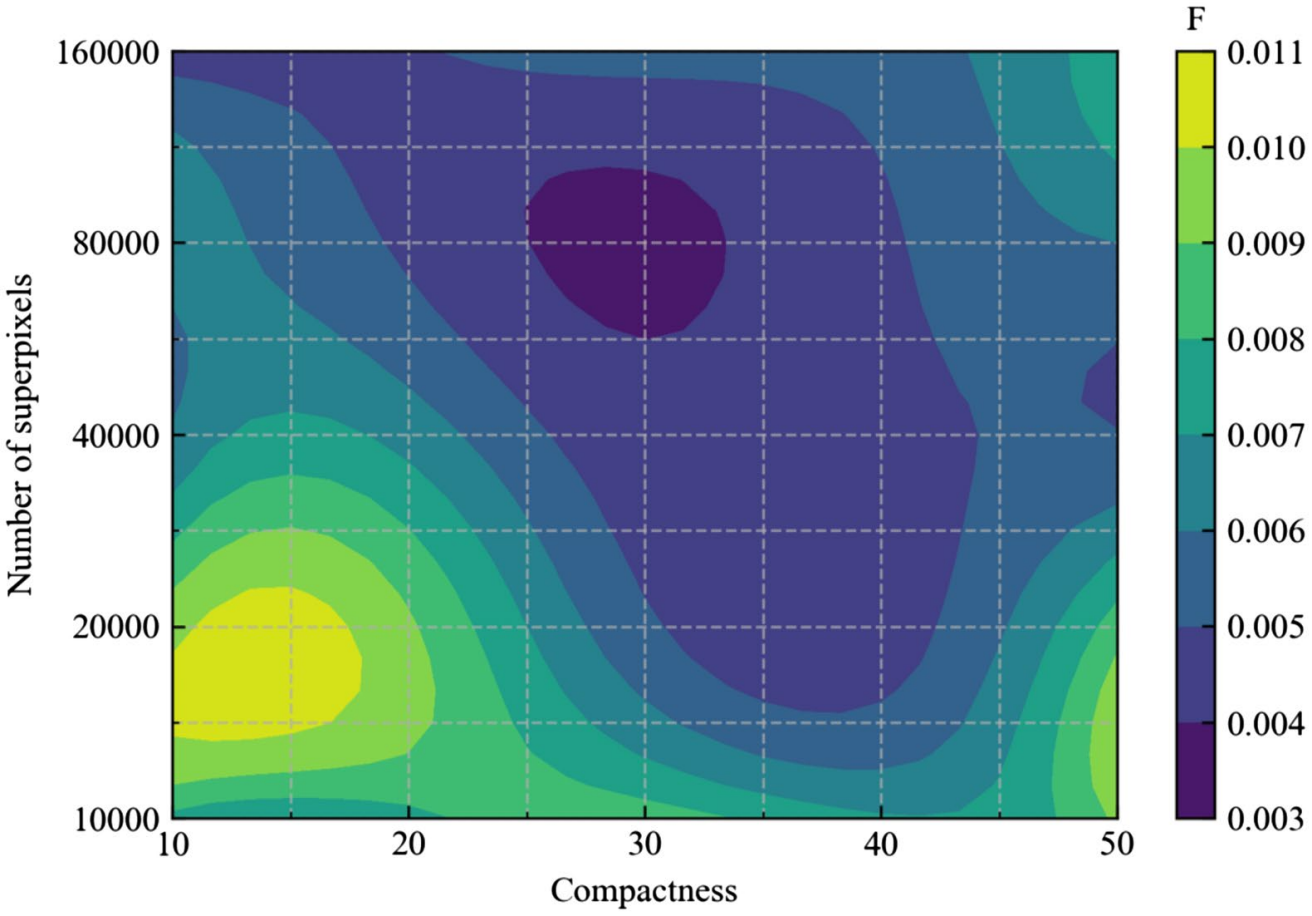
Contour map of F as a function of compactness values and numbers of superpixels.

As with the case of the number of classes, hyperparameters greatly affect the computation result. In this section, the effects of the compactness and the number of superpixels, which are essentially adjusted in superpixel segmentation processes, are investigated using the $F$ criterion. The microstructure given in Fig. 6a is used as an input image and the number of classes are set as three. The segmentation is repeated 10 times for each pair of hyperparameters and the average $F$ is taken. Figure 11 shows the dependence of $F$ on the compactness and the number of superpixels. It is commonly observed that $F$ decreases to a certain extent and then increases with increasing compactness. Since the compactness has a trade-off with the color similarity and spatial proximity, a very high or low compactness makes an algorithm yield superpixels based on limited information, resulting in failure to detect the region boundary^34^. In terms of the number of superpixels, a similar tendency is shown that a very high or low number reduces the quality of segmentation; extreme values for the number of superpixels result in the incorrect segmentation of pixels^35–37^. In conclusion, avoiding very high or too low values for the compactness and number of superpixels will provide a better segmentation result.

## Conclusion

We demonstrated the segmentation of the microstructure of a low-carbon steel without labeled images using a deep learning method. Specifically, CNN and the SLIC superpixel algorithm were introduced for pixelwise segmentation. Various microstructure images of steel composed of ferrite, pearlite, bainite, and martensite are used and regions of constituent phases were well distinguished. In addition, the quality of the segmentation results was assessed on the basis of various unsupervised segmentation evaluation criteria. We found that the $F$ criterion shows better performance than the $F{^{\prime}}$ and $Q$ criteria for the segmentation of the steel microstructure. Finally, the effect of hyperparameters on the segmentation was investigated and it was found that medium values are desirable for good performance. It is concluded that the deep-learning-based approach is efficient and fast method in distinguishing various microstructural features of low-carbon steels without the need to create labeled images.

## Data Availability

Source code of the algorithm described in this paper is available upon request.
