# Author Correction: Unsupervised microstructure segmentation by mimicking metallurgists’ approach to pattern recognition

**DOI:** 10.1038/s41598-021-88173-z

**Published:** 2021-04-14

**Authors:** Hoheok Kim, Junya Inoue, Tadashi Kasuya

**Affiliations:** 1 Institute for Industrial Science, The University of Tokyo, 4-6-1 Komaba, Meguro, Tokyo, 153-0041 Japan; 2 Graduate School of Engineering, The University of Tokyo, 7-3-1 Hongo, Bunkyo, Tokyo, 113-8656 Japan

Correction to: *Scientific Reports* 10.1038/s41598-020-74935-8, published online 20 October 2020

The Data Availability section is incomplete in the original Article. The Data Availability section should read:

‘Source code of the algorithm described in this paper is available from the URL below: https://github.com/inouejunyalab/for_public/tree/main/cnn_superpixel.’

